# Transient Global Amnesia Following Dobutamine Stress Echocardiography

**DOI:** 10.7759/cureus.84357

**Published:** 2025-05-18

**Authors:** Maryam Hanser, Diana Boyrazyan, Charisse N Chih, Antonio K Liu

**Affiliations:** 1 Internal Medicine, Adventist Health White Memorial, Los Angeles, USA; 2 Molecular and Cell Biology, University of California Berkeley, Berkeley, USA; 3 Neurology, Adventist Health White Memorial, Los Angeles, USA; 4 Neurology, Loma Linda University School of Medicine, Loma Linda, USA

**Keywords:** adrenergic stress, dobutamine stress echocardiography, hippocampal dysfunction, hippocampal hypoperfusion, transient global amnesia

## Abstract

Transient global amnesia is an uncommon neurological phenomenon characterized by sudden-onset anterograde amnesia, often accompanied by repetitive questioning and transient cognitive dysfunction, which resolves completely within 24 hours. While self-limiting, this can cause significant distress to both patients and their families, particularly when it arises in the context of medical interventions. Dobutamine stress echocardiography, a widely employed non-invasive diagnostic modality for assessing myocardial ischemia, valvular pathology, and left ventricular function, is generally regarded as safe and well-tolerated. There are only a few cases of transient global amnesia after dobutamine stress echocardiography. Our case report may align with the current belief that the adrenergic and hemodynamic stress induced by dobutamine stress echocardiography can occasionally elicit transient global amnesia in predisposed patients, challenging its reputation as a low-risk diagnostic procedure.

## Introduction

Transient global amnesia (TGA) is characterized by a distinctive sequence of sudden-onset anterograde amnesia, succeeded by retrograde amnesia. Specifically, individuals experiencing TGA manifest an abrupt, complete inability to formulate new memories while retaining alertness, self-awareness, and other cognitive functions. The impairment in new information retention endures for a brief period, typically seconds, while subsequent retrograde amnesia spans several hours to beyond days. The patient will often present with repetitive, stereotyped questions approximately every 30 seconds, querying information that has already been provided [1]. The proposed diagnostic criteria for TGA encompass the absence of both seizures and head injury, along with resolution within 24 hours. Additional criteria include the attack being witnessed, dysfunction limited to repetitive queries and amnesia, no other major neurologic signs or symptoms, no clouding of consciousness/cognitive defect/loss of personal identity, and transient memory loss [2]. Despite its dramatic presentation, TGA remains poorly understood, with an annual incidence of approximately three to eight cases per 100,000 individuals, with a heightened risk observed in those above 50 years of age. The average age of onset is 62 years, with a slight predilection for occurrence in men. Examining the recorded cases reveals episode durations ranging from two to 12 hours, with an average duration of six hours.

Patients with cardiovascular risk factors align with the demographic most susceptible to TGA. Episodes may be preceded by physical or mental shock, as well as extreme exertion; however, a significant portion of cases remain idiopathic. At present, a comprehensive pathophysiological explanation for TGA remains elusive. Some proposed mechanisms include transient ischemia, venous congestion, and neurohormonal dysregulation, particularly in the hippocampal regions critical for memory encoding and retrieval. None have become a generally accepted theory. Radiological examination of certain patients' MRIs reveals transient punctate lesions predominantly localized in the hippocampus or adjacent structures, with a predilection for the left hemisphere over the right or bilaterally. These lesions exhibit delayed and transient appearances, typically emerging 12 to 48 hours post-episode [1]. Some suggest the sensitivity is as high as 46% in a three Tesla MRI machine and goes up to 85% in a seven Tesla machine [3]. While self-limiting, TGA can cause significant distress to both patients and their families, particularly when it arises in the context of medical interventions. Therefore, it is important for practitioners to have a basic understanding of this entity. 

Dobutamine stress echocardiography (DSE), a widely employed non-invasive diagnostic modality for assessing myocardial ischemia, valvular pathology, and left ventricular function, is particularly important in patients who are incapable of exercising on a treadmill or stationary bike. Dobutamine, which mimics the effect of exercise by increasing heart rate and contractility, is administered to patients. Images of the heart are taken at baseline and simultaneously during increasing doses of dobutamine to assess cardiac function. A normal test shows increased and uniform heart muscle contraction, while an abnormal test shows reduced wall motion and is suggestive of ischemia secondary to blocked coronary arteries. Vital signs, including heart rate and blood pressure, are continuously monitored [4]. Some examples of absolute contraindications of DSE include patients with acute myocardial infarction within 48 hours, symptomatic severe aortic stenosis, uncontrolled arrhythmias, unstable angina, severe arterial hypertension, and decompensated or unstable heart failure with an ejection fraction less than 35%. DSE remains an essential diagnostic modality because it allows for the evaluation of ischemia for patient populations that are unable to complete an exercise treadmill test or if that test is not available due to resources. It is generally regarded as safe and well-tolerated, even taking into account palpitations or arrhythmias that may occur.

Known precipitants of TGA include psychological stress, vigorous physical activity, and medical procedures. Among these, cardiac stress testing is an established but rare trigger; there are only a few cases found in the literature. DSE, a non-invasive diagnostic test that simulates exercise by pharmacologically increasing heart rate and myocardial oxygen demand, is widely regarded as safe. However, its hemodynamic and adrenergic effects may predispose certain patients to transient brain (in particular hippocampal) dysfunction, culminating in TGA. Although cases of DSE-provoked TGA are infrequent, they highlight the necessity of vigilance, particularly in individuals with preexisting cerebrovascular or metabolic susceptibilities.

We present the case of an 81-year-old woman with multiple comorbidities who developed TGA immediately following a DSE. The temporal relationship between DSE and TGA in this patient may have revealed the hippocampus’s susceptibility to transient ischemia, vascular compromise, and adrenergic metabolic effects during such procedures. We did a literature search for similar cases and tried to establish the underlying etiologies.

## Case presentation

An 81-year-old Filipina with hypertension, type II diabetes mellitus, dyslipidemia, chronic kidney disease (stage II), and a history of transient ischemic attacks (TIA) presented with chest pain suggestive of cardiac ischemia. She has no history of migraine or seizure. Her regular home medications included clopidogrel, amlodipine, hydralazine, and losartan. She took her medication in the morning on the day of the test. She had undergone left knee arthroplasty one week earlier and was prescribed tramadol for postoperative pain. Her last use of tramadol was two days prior to the test. Upon admission, her electrocardiogram (ECG) and cardiac biomarkers were normal. A DSE was performed to evaluate for inducible myocardial ischemia. Her pre-infusion blood pressure was 165/80. During the DSE, the patient’s rhythm and blood pressure were monitored continuously. The target heart rate was achieved without the use of atropine. Her post-infusion heart rate was recorded as 93 with a blood pressure of 129/76. The examination was completed without complications, and the patient denied cardiac symptoms.

Approximately 30 minutes post-DSE, the patient developed sudden confusion and amnesia, which was witnessed by her daughter, a registered nurse. The patient repeatedly asked, “Where am I?” and “Why am I here?” while remaining oriented to her name and aware of her daughter’s presence. Her daughter reported that the patient had no recollection of recent events, including the reason for her hospitalization. Despite being reoriented constantly, the patient repeatedly posed the same questions every three to five minutes. Despite her cognitive impairment, the patient was alert, cooperative, and free of focal neurological deficits. 

**Figure 1:**
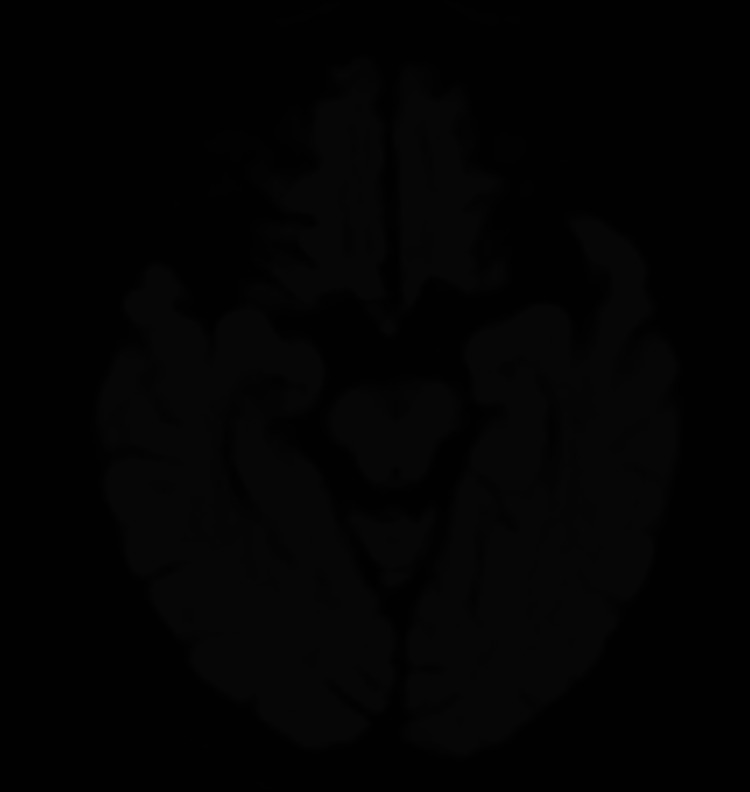
Negative MRI diffusion-weighted imaging (DWI) sequence at the level of mesial temporal lobes. No hippocampal abnormality noted. Study done 18 hours after symptom onset.

Neurology consultation revealed intact immediate registration of three objects, but zero out of three recall at the five-minute point. She had intact cranial nerve function, normal motor and sensory systems, and preserved reflexes. There was no altered level of consciousness. Her gait was at her usual state, and there was no urinary or bowel incontinence. A non-contrast CT of the head showed age-related involutional changes and chronic periventricular white matter ischemic lesions but no acute abnormalities. MRI, performed 18 hours after symptom onset, demonstrated no evidence of acute ischemic changes, particularly in the hippocampal regions (Figure 1). Carotid ultrasound revealed moderate bilateral atherosclerosis (50-69%) consistent with her cerebrovascular risk profile. An electroencephalogram (EEG) done 24 hours after symptom onset was negative for any epileptic waveform.

Laboratory studies included no pertinent positive findings or value. Transthoracic echocardiography (TTE) with bubble study revealed normal left ventricular systolic function (LVEF 68%), mild concentric left ventricular hypertrophy, and diastolic dysfunction. Pulmonary artery pressures were normal, and no intracardiac shunts were detected.

The patient’s symptoms resolved completely within six hours. By the following morning, she was fully oriented and had regained recall of events preceding her hospitalization. Her recall of three objects was intact at the 10-minute point. She was discharged with instructions for outpatient endocrinology and cardiology follow-up and remained neurologically asymptomatic at her six-week follow-up.

## Discussion

Our patient has classic symptoms of TGA. Her symptoms fulfilled all the criteria put forth by Hodges and Warlow [2]. The differential diagnosis was short and easy to discern, as there were no other physical findings, the MRI was negative, and symptoms rapidly resolved. There was no evidence of stroke. She had no history of migraine, and there was no headache. She had no history of seizures, and an EEG done on the next day was negative. Had an EEG been recorded during the patient’s symptomatic phase, it would have provided even stronger exclusion of epileptic activity. Unfortunately, we had no functional imaging capability.

A literature review identified two cases of TGA following DSE [5,6]. We attempted to draw similarities from past medical history and the patient’s background, but no common trend or common risk factors could be established. We then expanded our search to TGA after similar cardiac procedures and summarized below popular beliefs on current TGA etiologies; there are five possible etiologies besides stroke, seizure, or migraine: Valsalva, venous congestion, incompetent internal jugular valve; decreased blood flow to the hippocampus from the cardiac procedure; catecholamine or adrenergic overstimulation; stress, cortisol level elevation; and cortical spreading depolarization (CSD).

We explore the Valsalva/venous congestion/incompetent venous valve opinion first; as a matter of fact, both cases of TGA after DSE that we found attributed their case to this reason [5,6]. One case reported a 68-year-old woman with hypertension who experienced acute TGA immediately after a dobutamine-atropine stress echocardiogram, presenting with repetitive reassurance-seeking and impaired short-term memory. Onset was immediate, and symptoms lasted five hours. MRI, EEG, and neurological examination were otherwise unremarkable, and the patient made a complete recovery [5]. Another report described a 54-year-old woman who exhibited classical TGA symptoms during DSE two minutes after atropine injection. She had a remote medical history of deep vein thrombosis and was not on anticoagulation or other medications. Neuroimaging and laboratory evaluations were unremarkable, and she made a complete recovery the next day [6]. Both papers speculated that the reasons were decreased venous return from Valsalva, coupled with underlying incompetent jugular valves. Their supporting argument was derived from an earlier study on TGA and venous valve incompetence. In that study, 68% of TGA patients (as opposed to 33% of the controls) were found to have valvular incompetence [7].

Similarly, TGA can develop after other cardiac-related procedures, and those cases also found Valsalva and venous congestion as a plausible explanation. A 63-year-old woman underwent transesophageal echocardiography (TEE) without sedation for a pulmonary issue and developed TGA immediately after the extubation of the scope. Workup, MRI, and EEG were all negative, and the patient made a complete recovery the next day. Retching from the irritation of equipment was thought to be the trigger of reduced venous return [8]. In another patient, a 54-year-old woman with a history of TGA six months before. She had an “unremarkable medical history” and underwent TEE for acute chest pain and developed TGA immediately after the Valsalva maneuver following the intravenous injection of agitated saline. That led to the prompt termination of the cardiac testing. MRI and EEG were negative, and neurological examination was otherwise negative; her recovery was complete by 24 hours. Valsalva leading to a retrograde flow pattern in the internal jugular vein was implicated as the cause [9].

Transient cerebral hypoperfusion represents another plausible etiology. A 27-year-old man developed TGA immediately after radiofrequency catheter ablation of symptomatic supraventricular tachycardia. No medical history or medication list was disclosed. TGA was recognized by the family after the patient returned to the ward, and it lasted for six hours. Neurological examination was otherwise negative, and MRI had no diffusion-weighted imaging (DWI) abnormality; no EEG was performed. Rapid ventricular rate (220 per min), which led to transient cerebral ischemia, was their speculation [10]. In another case, a 71-year-old man with chronic obstructive pulmonary disease developed TGA minutes after successfully completing an incremental exercise test on a cycle ergometer. Detailed workup was not available. They suggested that transient ischemia secondary to altered vascular tone in the vertebrobasilar vessels was the reason [11]. A recent study performed on 48 healthy individuals without cardiovascular or cerebrovascular disease demonstrated significantly lower cerebral blood flow on MRI during the dobutamine-stress test [12]. None of their subjects developed any symptoms or TGA, and no pathological mechanism was proposed. One study has observed significant hippocampal hypoperfusion on single photon emission computed tomography (SPECT) imaging during the acute phase of TGA, and the restoration of memory was paralleled by the restoration of blood flow [13].

Proponents of the adrenergic overstimulation model have drawn parallels between TGA and Takotsubo cardiomyopathy, another stress-induced reversible syndrome. A case report documented the sudden onset of TGA in a 64-year-old man during a funeral. MRI and EEG were negative. Cardiological workup revealed dyskinesia of the left ventricular posterior, posterolateral, and apical parts of the left ventricular myocardium and apical ballooning. Neurological symptoms resolved within 24 hours, followed by cardiac symptoms over four days. This same report cited nine other cases that had been reported by 2019. It is interesting to note that nine out of 10 patients were female. Their age range was 57 to 72. Associated events were found to be four deaths, three stressful situations, one assault, one vigorous exercise (swimming), and one with an unknown trigger. Little common medical background was established. After the analysis, the authors believe catecholamine excess and overstimulation of cerebral adrenergic receptors in neurons led to pathophysiological changes within the brain, and memory deficit was the suggested etiology [14,15,16]. Dobutamine, an adrenergic agonist, may similarly precipitate TGA after DSE.

Stress, mediated by the stress hormone cortisol, has always been considered one of the triggers of TGA. A study of 20 TGA patients with 20 matched controls showed that hippocampus-dependent aversive learning processes showed impaired hippocampal responses on functional MRI with elevated cortisol levels after stress exposure [17]. Another study also looked at cortisol levels after cardiopulmonary exercise testing. Cortisol level was higher among 40 subjects and significant enough for the authors to suggest that cortisol level holds predictive value for cardiac disease severity and prognosis [18]. To tie cortisol level and TGA together, a study in 2019 found enhanced, elevated cortisol levels in all 14 subjects with TGA [19]. This can certainly directly tie in a patient with TGA after DSE.

From an electrophysiological standpoint, CSD has long been considered a possible mechanism for TGA [20]. CSD is usually affiliated with the aura phase of migraine headache; waves of hyperactivity followed by inhibition spreading outward from the occipital lobe have been seen on functional imaging during the visual aura of migraine. CSD affecting the hippocampal area, especially the rich CA3 recurrent collateral projections, has been thought to be a trigger for TGA [21]. The link between DSE and CSD may lie with the observation that adrenergic inhibition facilitates normalization after CSD, possibly offering neuroprotection [22]. In another study, a similar conclusion was reached that adrenergic receptor antagonism induces neuroprotection and facilitates recovery from CSD [23]. Adrenergic stimulation may have triggered or enhanced CSD and contributed to TGA.

After analyzing our case and selected publications, it is clear that the pathological mechanism of TGA after DSE is far from firmly established. The transient dysfunction of the hippocampus may be the final manifestation of different pathways. Although these pathways discussed above may seem unrelated, they are probably not mutually exclusive. Valsalva, venous congestion, decreased cerebral blood flow, adrenergic overstimulation, cortisol elevation, and CSD are all possibly present and related to each other to different degrees. The final presentation may also depend on the susceptibility of the patient. Given the absence of consistent risk factors or common past medical history, preprocedural screening is challenging and not practical. For example, it is not plausible to screen patients for venous valvular incompetence before undergoing DSE. Although DSE is still considered a relatively low-risk procedure, and TGA is still a relatively rare and poorly understood entity, TGA is increasingly being recognized as a possible complication.

## Conclusions

Our case and the few cases in the literature highlight the vulnerability of the hippocampus to rapid hemodynamic, neurohormonal, electrophysiological, or cerebrovascular changes during cardiac interventions. They also highlight the importance of recognizing TGA as a potential complication of stress testing to facilitate accurate diagnosis and management. Although self-limiting, TGA’s dramatic presentation may result in unnecessary testing and distress (for patient, family, and staff) if not promptly identified. This case contributes to the expanding, albeit limited, literature on TGA as a procedural complication of DSE. Further studies are needed to better understand the mechanisms underlying TGA and to develop possible screening strategies for at-risk populations undergoing cardiac stress testing or similar procedures.
